# A variable heart rate multi-compartmental coupled model of the cardiovascular and respiratory systems

**DOI:** 10.1098/rsif.2023.0339

**Published:** 2023-10-18

**Authors:** Sam Lishak, Gevik Grigorian, Sandip V. George, Nicholas C. Ovenden, Rebecca J. Shipley, Simon Arridge

**Affiliations:** ^1^ Department of Computer Science, University College London, London WC1E 6BT, UK; ^2^ Department of Mechanical Engineering, University College London, London WC1E 6BT, UK; ^3^ Department of Mathematics, University College London, London WC1E 6BT, UK

**Keywords:** cardiovascular system, respiratory system, variable heart rate, ordinary differential equations

## Abstract

Current mathematical models of the cardiovascular system that are based on systems of ordinary differential equations are limited in their ability to mimic important features of measured patient data, such as variable heart rates (HR). Such limitations present a significant obstacle in the use of such models for clinical decision-making, as it is the variations in vital signs such as HR and systolic and diastolic blood pressure that are monitored and recorded in typical critical care bedside monitoring systems. In this paper, novel extensions to well-established multi-compartmental models of the cardiovascular and respiratory systems are proposed that permit the simulation of variable HR. Furthermore, a correction to current models is also proposed to stabilize the respiratory behaviour and enable realistic simulation of vital signs over the longer time scales required for clinical management. The results of the extended model developed here show better agreement with measured bio-signals, and these extensions provide an important first step towards estimating model parameters from patient data, using methods such as neural ordinary differential equations. The approach presented is generalizable to many other similar multi-compartmental models of the cardiovascular and respiratory systems.

## 1. Introduction

Computational models of biological systems can help further understanding of physiology, and drive the development of predictive tools for clinical decision-making. A wide variety of models of the human cardiovascular system (CVS) have been proposed, and their complexity is frequently classified in terms of the number of spatial dimensions that are simulated [1,2]. At the simplest end of the scale lie zero-dimensional (lumped parameter) models, which have no spatial information and instead model the CVS as discrete compartments (also known as a pressure–volume or PV model). One-dimensional models permit estimation of how flow and pressure waves propagate through a network of blood vessels [3–5]. Two-dimensional, or more commonly, three-dimensional models use finite-element analysis (FEA) and/or computational fluid dynamics (CFD) to obtain highly detailed simulations of fluid flows [6,7]; these often still rely on lower dimensional models for the boundary conditions.

This work is concerned only with zero-dimensional time-dependent ordinary differential equation (ODE) models. Early examples of these models are the mono-compartment Windkessel [8,9] and Westkessel [10,11] models, employing electrical circuit analogies. By comparison, multi-compartment models split the CVS into discrete segments, enhancing the level of detail; using more compartments, however, requires more parameters to be estimated. One of the more extreme examples of this is the so-called Guyton model [12], which describes most of the main blood vessels as well as the regulation of autonomous and hormone systems. More commonly, simpler representations are used where the complexity can be increased in an area of interest [2,13,14]. These models are typically only analysed once they reach a stable orbit, where the system returns to the same state after each heartbeat. As a result, such models are typically only simulated for as long as required for the initial transient behaviour (due to imprecise or unknown initial conditions) to settle; beyond this, no extra information can be obtained by running for longer.

The activity of the CVS and the respiratory system are strongly coupled, in part due to the location of the heart and adjacent arteries and veins inside the thoracic cavity, which experiences varying pressure due to activity of the respiratory muscles. Including these effects leads to more detailed models capable of investigating specific situations, such as the Valsalva manoeuvre or artificial ventilation, over longer time periods [2,15–21].

These models may include some sort of regulatory system model for the control of heart rate (HR) and/or respiratory rate, leading to greatly increased model complexity [19,21–25]. Although this is useful for building understanding of specific conditions, implementing *in silico* all possible pathways by which respiration and circulation can be controlled *in vivo* is infeasible; for example, emotional responses can significantly affect HR, yet a model aiming to quantify or predict this in a clinical setting would be very challenging to design due to the plethora of underpinning mechanisms (and their quantification) that would be required.

Some other contemporary CVS models, for example CircAdapt [26–28], permit open-loop control of HR, although it can only be changed in discrete steps and requires the ODE integration to be restarted at each change point (typically between predetermined ‘rest’ and ‘exercise’ settings).

This paper focuses on Smith’s multi-compartment model of the human CVS with ventricular interaction [14,29], along with Jallon’s extension to this model to simulate interaction between the CVS and the respiratory system [18]. These models are designed to minimize complexity, and do not contain any mechanism for dynamic variation of HR. All models were implemented using JAX [30,31], with a view to permitting autodifferentiation of the ODE solutions. Future implications of this work, for example grey-box modelling, are discussed in §5.

Two novel modifications to these models are presented and explored in this paper. Firstly, a correction is derived in §2.3 to stabilize the behaviour of Jallon’s respiratory system model over longer simulation times. Secondly, an extension to the cardiac driver function in the CVS model is developed in §2.4 to allow simulation incorporating a variable HR as an arbitrary function of time.

After specifying realistic model parameters and initial conditions in §2.5, results from simulations of the various models (including those previously published as well as those incorporating the extensions developed here) are presented in §3. The results demonstrate stable long-term cyclical behaviour of the interactive system even under variable HR conditions. A discussion follows in §4.

The approach to implementing variable HR developed herein is in theory generalizable to many other ODE models; the model given here is just one example of this. In §5, the paper concludes by discussing this, and also some further opportunities for applications of such models.

## 2. Methods

A review of Smith’s model of the human CVS [14] is included in §2.1, along with an extension by Jallon [18] to simulate interaction between the heart and lungs in §2.2. Subsequently, §2.3 presents how Jallon’s model can be stabilized to allow long-term simulation, and a method for implementing variable HR is developed in §2.4. Finally, §2.5 contains a complete listing of model parameters and initial conditions.

Python (JAX) implementations of the models described below are available on GitHub at slishak/cvsx.

### 2.1. Cardiovascular system model

**Figure 1.**
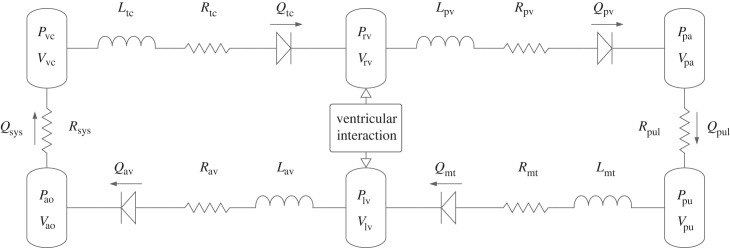
Schematic of the full cardiovascular system model. The inductances are only present when modelling inertial flows. The diodes represent check valves. Nomenclature is defined in tables 1 and 2.

The CVS has been minimally modelled as six compartments connected in a closed loop by valves, resistances and optionally inductances [14,22,32]. A schematic is given in figure 1. This model has been widely applied in human and animal studies [1]. It takes into account interaction between the ventricles through the ventricular septum and the pericardium. There are two models of valve dynamics that can be used: a simple non-inertial check valve model which allows flow only during a negative (decreasing) pressure gradient, and a more realistic inertial model described as ‘open on pressure, close on flow’ which allows flow through the valve to continue due to the fluid inertia even if the pressure gradient becomes positive (increasing).

**Table 1.** Pressure–volume relationships for the non-inertial cardiovascular system [22], with modifications after Jallon [18] in brackets (which are not used in any of the simulations below, but retained for reference). *E*_es_ is the contractility, *V*_*d*_ is the compartment dead-space, *V*_0_ is the volume at zero pressure, and *P*_0_ and *λ* are the gradient and curvature respectively for the EDPVR.

parameter (units)	*E*_es_ (mmHg l^−1^)	*V*_*d*_ (l)	*V*_0_ (l)	*λ* (l^−1^)	*P*_0_ (mmHg)
left ventricle free wall (lvf)	3405	0.005	0.005	15	1.2751
right ventricle free wall (rvf)	653	0.005	0.005	15	1.2001
septum free wall (spt)	48754 (3750)	0.002	0.002	435 (35)	1.1101
pericardium (pcd)	—	—	0.2	30	0.5003
vena cava (vc)	11.25 (2)	2.83	—	—	—
pulmonary artery (pa)	337.5	0.16	—	—	—
pulmonary vein (pu)	6	0.2	—	—	—
aorta (ao)	705	0.8	—	—	—

**Table 2.** Other parameters for the non-inertial cardiovascular system [22], with modifications after Jallon [18] in brackets.

parameter	value
mitral valve resistance (*R*_mt_)	0.45 mmHg s l^−1^
aortic valve resistance (*R*_av_)	10.5 mmHg s l^−1^
tricuspid valve resistance (*R*_tc_)	1.35 mmHg s l^−1^
pulmonary valve resistance (*R*_pv_)	3.6 mmHg s l^−1^
pumonary circulation resistance (*R*_pul_)	142.5 mmHg s l^−1^
systemic circulation resistance (*R*_sys_)	1050 mmHg s l^−1^
heart rate (HR)	$80\;(54)\;\min^{-1}$
number of exponentials in cardiac driver (*N*)	1
cardiac driver scale ($A_{1},{\hat{A}}_{1}$)	1
cardiac driver width ($B_{1},{\hat{B}}_{1}$)	80
cardiac driver offset (*C*_1_)	30/80 (30/54) s
cardiac driver offset, variable HR (${\hat{C}}_{1}$)	0.5
total blood volume (*V*_tot_)	5.5 l
thoracic cavity pressure (*P*_pl_)	−4 mmHg

The model is described in full detail below, as there are a few cosmetic changes for readability compared with the referenced works (conditional statements are used instead of equivalent Heaviside functions). Each compartment’s volume is governed by an ODE.

#### 2.1.1. Single ventricle dynamics

**Figure 2.**
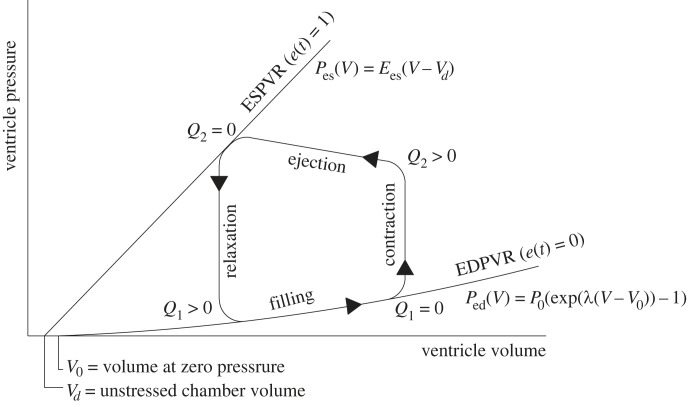
Example pressure–volume diagram for ventricular and septal walls (*V*_lvf_, *V*_rvf_, *V*_spt_) over the cardiac cycle. © 2007 Elsevier Science & Technology Journals, from [22]; permission for use conveyed through Copyright Clearance Center, Inc.

The function of a ventricle in isolation is described by a PV diagram, which shows how the PV relationship changes through the cardiac cycle (figure 2). The two main characteristics are the end systolic pressure–volume relationship (ESPVR) and the end diastolic pressure–volume relationship (EDPVR). The ESPVR represents the maximum elastance of the ventricle at the end of systole (after contraction and ejection) and the EDPVR represents the minimum elastance during diastole (when the ventricle is relaxed and filling). Nomenclature of all variables and parameters in the equations below is given in tables 1 and 2, and all dimensional quantities are expressed in seconds, litres and mmHg except for HR which is expressed in min^−1^ by convention.

In this model, the equations governing the ESPVR and EDPVR, respectively, are given by

2.1 $$P_{\mathrm{es},m}(V_{m})=E_{\mathrm{es},m}(V_{m}-V_{d,m})$$

and

2.2 $$P_{\mathrm{ed},m}(V_{m})=P_{0,m}(e^{\lambda_{m}(V_{m}-V_{0,m})}-1),$$

where the subscript *m* specifies the virtual volume in accordance with the interactions between the ventricles and septum, as described below in §2.1.2 [14]. The full PV relationship applying to a ventricle, or time-varying elastance [33], is then given by

2.3 $$P_{m}(V_{m},t)=e(t)P_{\mathrm{es},m}(V_{m})+(1-e(t))P_{\mathrm{ed},m}(V_{m})$$

2.4 $$\mathrm{where}\;e(t)=\sum_{i=1}^{N}A_{i}\;e^{-B_{i}{\left((t\mathrm{mod}(60/\mathrm{HR}))-C_{i}\right)}^{2}}.$$

**Figure 3.**
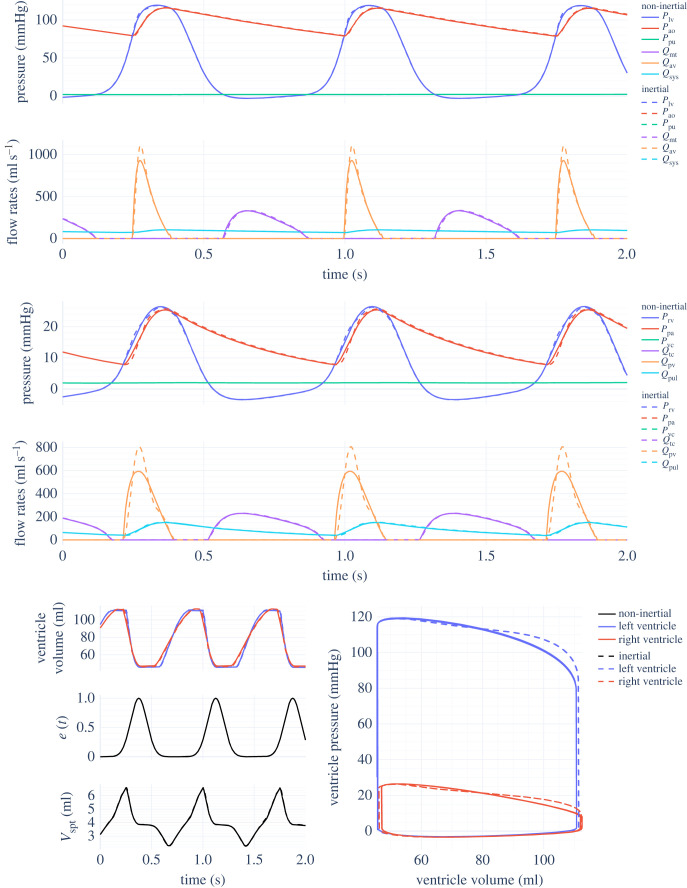
Smith non-inertial and inertial CVS model outputs with parameters from tables 3 and 4, providing a like-for-like comparison on the effects of including inertia in the model.

The function *e*(*t*) (equation (2.4)) is known as the cardiac driver function, which varies between 0 (diastole) and 1 (systole) over each cardiac cycle and is continuous and periodic with period 60/HR s; the multiplication by 60 is because HR is conventionally expressed in beats per minute, but *t* has units of seconds. A wide variety of such functions have been proposed, with complexities ranging from simple sums of Gaussian terms [34,35] to data based on invasive measurements of the CVS [36]. The version presented in equation (2.4) is a clarified version of that from Smith’s original model [14], with explicit modulo wrapping of *t* [1]. Furthermore, note that when *N* = 1 (as commonly used), *A*_1_ = 1 to satisfy 0 ≤ *e*(*t*) ≤ 1 and *C*_1_ = 30/HR s (half a cardiac period) to satisfy continuity, leaving *B*_1_ and HR as the only free parameters. A visualization of this choice of *e*(*t*) is shown in the bottom left of figure 3. An extension to the above to allow for variable HR is defined in §2.4.

**Table 3.** Pressure–volume relationships for the inertial cardiovascular system [1]. Parameters marked with (*) differ from the source, for reasons explained in the main text.

parameter	*E*_es_ (mmHg l^−1^)	*V*_*d*_ (l)	*V*_0_ (l)	*λ* (1/l)	*P*_0_ (mmHg)
left ventricle free wall (lvf)	2879.8	0	0	33	0.1203
right ventricle free wall (rvf)	585	0	0	23	0.2157
septum free wall (spt)	48 754	0.002	0.002	435	1.1101
pericardium (pcd)	—	—	0.2	30	0.5003
vena cava (vc)	5.9	0	—	—	—
pulmonary artery (pa)	369	0	—	—	—
pulmonary vein (pu)	7.3	0	—	—	—
aorta (ao)	691.3 (*)	0	—	—	—

**Table 4.** Other parameters for the inertial cardiovascular system [1]. Parameters marked with (*) differ from the source, for reasons explained in the main text. The cardiac driver and thoracic pressure parameters are as table 2.

parameter	value
mitral valve (*R*_mt_, *L*_mt_)	15.8 mmHg s l^−1^, 7.6968 × 10^−2^ mmHg s^2^ l^−1^
aortic valve (*R*_av_, *L*_av_)	18 mmHg s l^−1^, 1.2189 × 10^−1^ mmHg s^2^ l^−1^
tricuspid valve (*R*_tc_, *L*_tc_)	23.7 mmHg s l^−1^, 8.0093 × 10^−2^ mmHg s^2^ l^−1^
pulmonary valve (*R*_pv_, *L*_pv_)	5.5 mmHg s l^−1^, 1.4868 × 10^−1^ mmHg s^2^ l^−1^
pumonary circulation resistance (*R*_pul_)	155.2 mmHg s l^−1^ (*)
systemic circulation resistance (*R*_sys_)	1088.9 mmHg s l^−1^ (*)
total blood volume (*V*_tot_)	1.5 l (*)

#### 2.1.2. Ventricular interaction

**Figure 4.**
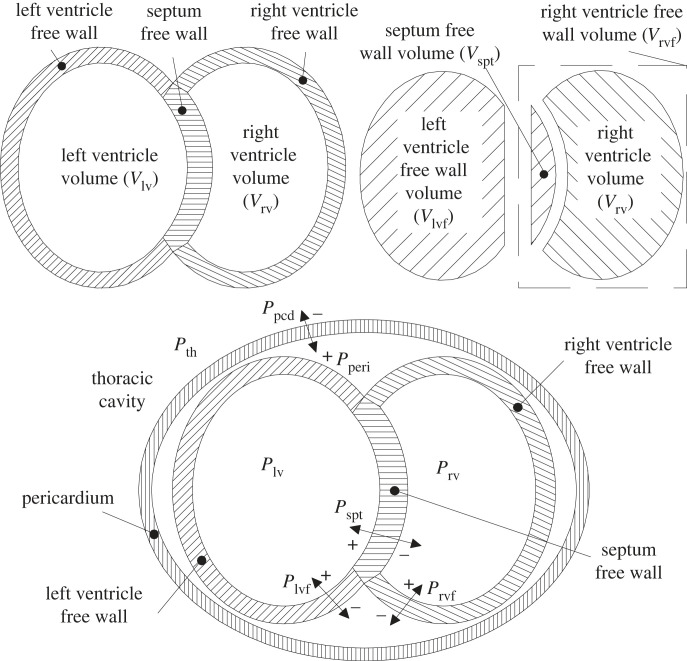
Diagram explaining how the left and right ventricles are split into three free wall volumes inside the pericardium. © 2005 Elsevier Science & Technology Journals, from [32]; permission for use conveyed through Copyright Clearance Center, Inc.

Due to the coupling between the two ventricles, rather than simulate them individually with equation (2.3), three virtual volumes are used: the left and right ventricle free wall volumes (*V*_lvf_ and *V*_rvf_), and the septum free wall volume (*V*_spt_). These are related to the actual left/right ventricle volumes as shown in figure 4 and equations (2.5) and (2.6) below. The pericardium volume *V*_pcd_ is the sum of the left and right ventricle volumes, or equivalently the sum of the ventricle free wall volumes (equation (2.7)), thus

2.5 $$V_{\mathrm{lvf}}(t)=V_{\mathrm{lv}}(t)-V_{\mathrm{spt}}(t),$$

2.6 $$V_{\mathrm{rvf}}(t)=V_{\mathrm{rv}}(t)+V_{\mathrm{spt}}(t)$$

2.7 $$\mathrm{and}\;\;V_{\mathrm{pcd}}(t)=V_{\mathrm{lv}}(t)+V_{\mathrm{rv}}(t)=V_{\mathrm{lvf}}(t)+V_{\mathrm{rvf}}(t).$$

The corresponding pressures are then calculated with equation (2.3) for *m* = lvf, rvf and spt, respectively. Because the pericardium sits inside the thorax, the thoracic (pleural) pressure *P*_pl_ is also applied (equation (2.8)), which is a constant in this model (but later becomes time-varying in §2.2 when covering Jallon’s model). Similarly, the ventricles sit inside the pericardium so the pericardium pressure *P*_peri_(*t*) is applied to equations (2.9) and (2.10). The septal pressure is just the difference between the left and right ventricle pressure (equation (2.11)). Hence,

2.8 $$P_{\mathrm{peri}}(t)=P_{\mathrm{pcd}}(V_{\mathrm{pcd}}(t),t)+P_{\mathrm{pl}},$$

2.9 $$P_{\mathrm{lv}}(t)=P_{\mathrm{lvf}}(V_{\mathrm{lvf}}(t),t)+P_{\mathrm{peri}}(t),$$

2.10 $$P_{\mathrm{rv}}(t)=P_{\mathrm{rvf}}(V_{\mathrm{rvf}}(t),t)+P_{\mathrm{peri}}(t)$$

2.11 $$\mathrm{and}\;P_{\mathrm{spt}}(V_{\mathrm{spt}}(t),t)=P_{\mathrm{lvf}}(V_{\mathrm{lvf}}(t),t)-P_{\mathrm{rvf}}(V_{\mathrm{rvf}}(t),t).$$

To complete the system of equations, the septum volume *V*_spt_(*t*) must be found such that equations (2.3), (2.5), (2.6) and (2.11) are all satisfied. This does not have an analytic solution, but can be solved using a nonlinear root-finder applied to the resulting residual function,

2.12 $$\begin{array}{rl} \;f\;(V_{\mathrm{spt}}) & =P_{\mathrm{lvf}}(V_{\mathrm{lv}}(t)-V_{\mathrm{spt}},t)\\ & \;-P_{\mathrm{rvf}}(V_{\mathrm{rv}}(t)+V_{\mathrm{spt}},t)-P_{\mathrm{spt}}(V_{\mathrm{spt}},t). \end{array}$$

The gradient *f*′(*V*_spt_) is also simple to compute by autodifferentiation or hand-calculation, which can be used for more efficient optimization (for example, using the Newton–Raphson algorithm). The uniqueness of the solution of *f*(*V*_spt_) = 0 can be verified by observing from equation (2.3) that d*P*_*m*_/d*V*_*m*_ > 0 for any set of physical (positive) parameter values, and therefore that *f*′(*V*_spt_) is negative for any *V*_spt_ value (and so *f*(*V*_spt_) cannot cross zero more than once).

Note that linear approximations to the system above have been proposed [18,37], but they have generally been found here to be unnecessary if root finder is initialized with the solved *V*_spt_ from the last accepted ODE step, as the Newton–Raphson algorithm then converges to an acceptable tolerance within one or two iterations. However, when trying to backpropagate gradients through the ODE solution [38], specialized solvers [31,39] may be required. Furthermore, this backpropagation through a nonlinear solver can represent an additional slowdown. With this in mind, Jallon’s EDPVR linearization [18], given by

2.13 $$P_{\mathrm{ed},\mathrm{lin},\mathrm{spt}}(V)=P_{0,\mathrm{spt}}\lambda_{\mathrm{spt}}(V-V_{0,\mathrm{spt}}),$$

replaces equation (2.2) only in the solution for *V*_spt_, yielding

2.14 $$V_{\mathrm{spt}}=\frac{e(t)(P_{\mathrm{es},\mathrm{lvf}}(V_{\mathrm{lv}})-P_{\mathrm{es},\mathrm{rvf}}(V_{\mathrm{rv}})+E_{\mathrm{es},\mathrm{spt}}V_{d,\mathrm{spt}})\;+\;(1-e(t))(P_{\mathrm{ed},\mathrm{lin},\mathrm{lvf}}(V_{\mathrm{lv}})-P_{\mathrm{ed},\mathrm{lin},\mathrm{rvf}}(V_{\mathrm{rv}})+\lambda_{\mathrm{spt}}P_{0,\mathrm{spt}}V_{0,\mathrm{spt}})}{e(t)(E_{\mathrm{es},\mathrm{lvf}}+E_{\mathrm{es},\mathrm{rvf}}+E_{\mathrm{es},\mathrm{spt}})\;+\;(1-e(t))(\lambda_{\mathrm{lvf}}P_{0,\mathrm{lvf}}+\lambda_{\mathrm{rvf}}P_{0,\mathrm{rvf}}+\lambda_{\mathrm{spt}}P_{0,\mathrm{spt}})},$$

the full form of which has not been explicitly given before. In equation (2.14), the (*t*) argument has been hidden from *V*_spt_(*t*), *V*_lv_(*t*) and *V*_rv_(*t*) to reduce clutter. This optional linearization can result in substantially different behaviour, shown later in this paper.

#### 2.1.3. Valve dynamics

Neglecting inertial effects, the equation for the instantaneous flow rate *Q*_*v*_(*t*) induced within an internal vasculature *v* of resistance *R*_*v*_ from an upstream pressure *P*_up,*v*_(*t*) to a downstream pressure *P*_down,*v*_(*t*) is given by

2.15 $$Q_{v}(t)=\frac{P_{\mathrm{up},v}(t)-P_{\mathrm{down},v}(t)}{R_{v}},$$

for *v* = pv, pul, mt, av, sys and tc, and the upstream/downstream pressures correspond to those shown in figure 1 for vasculature *v*. When a simple non-inertial valve is introduced, limiting the flow through *v* to one direction only, a ramp function (equation (2.17)) is applied to the flow rate such that it is never negative,

2.16 $$Q_{v}(t)=r\left(\frac{P_{\mathrm{up},v}(t)-P_{\mathrm{down},v}(t)}{R_{v}}\right)$$

and

2.17 $$r(x)=\left\{ \begin{array}{cc} x,\; & x\geq 0\;\\ 0,\; & \text{otherwise.}\; \end{array}\right.$$

When considering inertia, the flow rate through a valve becomes an ODE state, with a derivative function given by

2.18 $$\frac{dQ_{v}}{dt}=\left\{ \begin{array}{cc} \frac{P_{\mathrm{up},v}(t)-P_{\mathrm{down},v}(t)-Q_{v}(t)R_{v}}{L_{v}}, & \begin{array}{c} Q_{v}(t)>0\;\mathrm{or}\;\;\\ P_{\mathrm{up},v}(t)>P_{\mathrm{down},v}(t) \end{array}\\ 0,\;\; & \text{otherwise}\;\; \end{array}\right.$$

where *L*_*v*_ is a constant inductance [40]. An ‘open on pressure, close on flow’ valve law is used. Note that as long as *Q*_*v*_(0) is positive, in theory the valve law as stated should prevent the flow rate ever becoming negative (as d*Q*_*v*_/d*t* can never be negative when *Q*_*v*_(*t*) = 0), but in practice, the tolerances in ODE solvers mean that there is typically some small constant negative flow rate; this is quoted as typically being between −1 × 10^−4^ and −1 × 10^−6^ [37] but depends on the choice of ODE solver used, the units of the states, and the error (step size) control. The model as presented here is equivalent to the full Heaviside formulation in [37].

Regardless of whether inertia is being considered, the rate of change volume of a single chamber *c* is then described by equation (2.19), assuming incompressible fluid: the rate of change of volume (mass) in the chamber is equal to the net inflow of volume (mass). To account for the tiny negative flow rate that is possible when considering inertia (equation (2.18)), the ramp function in equation (2.17) is used to prevent the error propagating around the rest of the model (although it is not strictly necessary with the non-inertial model, as it is already applied in equation (2.15)),

2.19 $$\frac{dV_{c}}{dt}=r(Q_{\mathrm{in},c})-r(Q_{\mathrm{out},c}),$$

for *c* = rv, pa, pu, lv, ao and vc, and the flow rates in/out correspond to those shown in figure 1 for compartment *c*.

The valve conditions shown in equations (2.17) and (2.18) are also suitable for use with the event-handling capabilities of some ODE solvers, by only switching from one branch to the other when an exact zero crossing of the condition is found. As this is not currently supported by the chosen ODE solver [31], this might be a future improvement, although the use of an adaptive step ODE solver already achieves a low error around this discontinuity [37,41].

#### 2.1.4. Full closed-loop model

The final model is formed by setting the flow rate out of each compartment in figure 1 equal to the flow rate into the next compartment. The implementation used to generate the results below is almost the same as [1,37] but with the pleural pressure *P*_pl_ applied to the pulmonary vein and artery pressures *P*_pa_ and *P*_pu_ [18].

The total blood volume,

2.20 $$\begin{array}{r} V_{\mathrm{tot}}(t)=V_{\mathrm{pa}}(t)+V_{\mathrm{pu}}(t)+V_{\mathrm{lv}}(t)+V_{\mathrm{ao}}(t)+V_{\mathrm{vc}}(t)+V_{\mathrm{rv}}(t), \end{array}$$

is conserved throughout the simulation, as it is a closed fluid flow system. The initial individual compartment volumes are less important, as they will not affect the asymptotic behaviour of the system as long as they are feasible. Stable initial compartment volumes can be found manually by running the model from a reasonable starting point (e.g. tables 6 or 7) and recording a set of state values from the orbit that the model converges to. Some other possible approaches are discussed in [29]. Initial flow rates for the inertial model can be found using equation (2.15) (i.e. assuming zero inertia).

### 2.2. Combining respiratory and cardiovascular models

The CVS model presented above stabilizes to periodic behaviour, with every heartbeat exhibiting the same dynamics. However, in reality, the pleural pressure *P*_pl_ is not constant but varies with the respiratory rate, which (among many other factors) causes beat-to-beat variation in the cardiovascular dynamics. In Jallon’s heart-lung model [18], the respiratory rate is controlled by the central respiratory pattern generator, which is based on the Liènard system [42] shown in equation (2.23); the system of ODEs then defined by

2.21 $$\frac{dx}{dt}=\alpha \left(\;f(x(t),y(t))-\mathrm{HB}\frac{dV_{\mathrm{alv}}}{dt}\right),$$

2.22 $$\frac{dy}{dt}=\alpha x(t)$$

2.23 $$\mathrm{and}\;\;\;f\;(x,y)=(ay^{2}+by)(x+y).$$

can be simulated, where *x*(*t*) is a hidden variable and *y*(*t*) is the pattern. *V*_alv_(*t*) is a state of the respiratory model (equation (2.28)). The term HB(d*V*_alv_/d*t*) refers to the Hering–Breuer reflex [43,44] which is meant to prevent over-inflation of the lungs.

The respiratory muscle pressure *P*_mus_(*t*) is then simulated following equation (2.24), where *λ* and *μ* are (non-physical) parameters,

2.24 $$\frac{dP_{\mathrm{mus}}}{dt}=\lambda y(t)+\mu .$$

The states *y*(*t*) and *P*_mus_(*t*) from the above system of equations feed into the passive mechanical respiratory system model, which is unchanged from [18] (except for the movement of the *P*_mus_ state to the central respiratory pattern generator model). The equations are defined below where *V*_bth_(*t*) (equation (2.25)) is the intrathoracic blood volume, and *V*_alv_(*t*) is the alveolar volume. The pleural pressure is given by equation (2.27). One more state is introduced, *V*_alv_(*t*), with a derivative defined by equation (2.28),

2.25 $$V_{\mathrm{bth}}(t)=V_{\mathrm{pcd}}(t)+V_{\mathrm{pu}}(t)+V_{\mathrm{pa}}(t),$$

2.26 $$V_{\mathrm{th}}(t)=V_{\mathrm{bth}}(t)+V_{\mathrm{alv}}(t),$$

2.27 $$P_{\mathrm{pl}}(t)=P_{\mathrm{mus}}(t)+E_{\mathrm{cw}}(V_{\mathrm{th}}(t)-V_{\mathrm{th},0})$$

2.28 $$\mathrm{and}\;\;\;\frac{dV_{\mathrm{alv}}}{dt}=-\frac{P_{\mathrm{pl}}(t)+E_{\mathrm{alv}}V_{\mathrm{alv}}(t)}{R_{ca}+R_{ua}}.$$

The full respiratory/cardiovascular model can be simulated as a system of ODEs, constructing a right-hand side function defining the state derivatives using the following procedure (after setting appropriate initial conditions and parameter values, discussed in §2.5):

(i) receive time *t* and states (CVS volumes and flow rates, *x*(*t*), *y*(*t*), *P*_mus_(*t*) and *V*_alv_(*t*)),
(ii) call the respiratory system model (equations (2.25)–(2.28)), yielding d*V*_alv_/d*t*,
(iii) call the respiratory pattern generator (equations (2.21)–(2.23)), yielding d*x*/d*t*, d*y*/d*t* and d*P*_mus_/d*t*,
(iv) call the CVS model (§2.1) with *P*_pl_(*t*) from equation (2.27) used in equation (2.8), thus obtaining all remaining state derivatives.

### 2.3. Correcting and stabilizing the respiratory model

**Figure 5.**
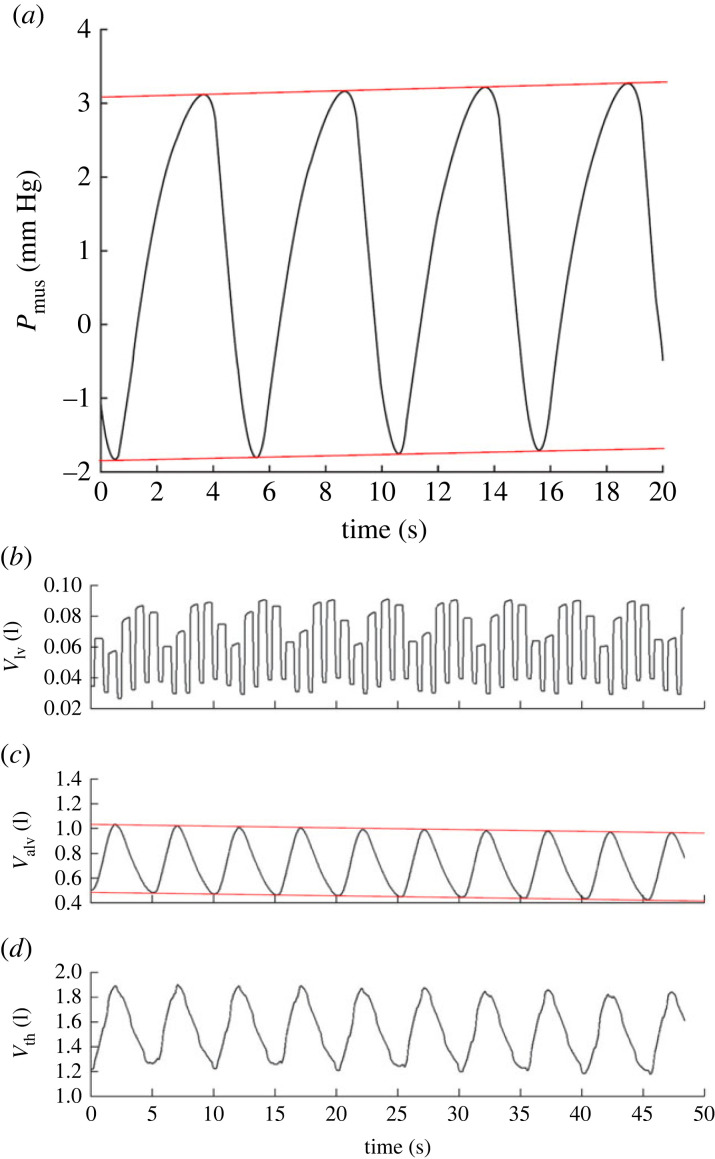
Plots of *P*_mus_ and *V*_alv_ from the Jallon heart-lung model, which show undesired non-stationary behaviour with *P*_mus_ steadily increasing and *V*_alv_ decreasing. © 2009 The Royal Society (UK), from [18]; permission for use conveyed through Copyright Clearance Center, Inc. (with overlaid red lines to accentuate the drift).

Equation (2.24) in §2.2 has been found to cause a gradual drift of *P*_mus_ over time, resulting in long-term instability of the solution and unbounded increase or decrease in alveolar volume *V*_alv_. This is visible in Jallon’s original paper (figure 5) and is also possibly the cause of issues from other referenced uses of this model [45]. A later paper from the same author introduces a slightly modified central respiratory pattern generator model, but this is understood to be a simplification in order to simulate a specific phenomenon rather than a general model improvement (and parameter unit inconsistencies further confuse the matter) [46].

Although equation (2.21) contains a term that supposedly prevents over-inflation of the lungs through the Hering–Breuer reflex, the justification for this implementation is unclear (an increase in *V*_alv_ acts to decrease the third derivative of *P*_mus_). Although these reflexes were historically thought to play a significant role in the regulation of ventilation, they are now understood to be largely inactive in adult humans (except while exercising), although there is some evidence that they may be more important in newborn babies [47]. Regardless, as the respiratory pattern generator is only required to synthesize realistic behaviour for *P*_mus_, there is little need for the complication of trying to mimic regulatory systems in the model (and furthermore, no clear reason for implementing just one element of a complicated control system).

Below, a modification is proposed to add an extra term to essentially perform integral feedback control (a type of proportional-integral-derivative or PID control [48]) on *P*_mus_, introducing a new parameter *β* in equation (2.29) (replacing equation (2.24)) which stabilizes the solution for small positive *β* (for example, *β* = 0.1 s^−1^). As this acts to prevent *P*_mus_ and *V*_alv_ steadily increasing or decreasing over time, the attempt to implement the Hering–Breuer reflex in equation (2.21) can be discarded (replacing it with equation (2.30)), thus

2.29 $$\frac{dP_{\mathrm{mus}}}{dt}=\lambda y(t)+\mu -\beta P_{\mathrm{mus}}(t)$$

and

2.30 $$\frac{dx}{dt}=\alpha f\;(x(t),y(t)).$$

### 2.4. Extending model to variable heart rates

Here, an original modification to the CVS model in §2.1 is discussed. The HR enters only as a fixed parameter in equation (2.4); if the model were to simulate intensive care unit (ICU) patient data, it would need to be able to have the HR varying with time, as this is an important feature of the physiology of patients suffering from multiple pathologies. This can be achieved by introducing another auxiliary state *s*(*t*) with a derivative defined by equation (2.31). The function HR(*t*) is an arbitrarily defined function of time; it could be a function which interpolates measured patient HR values, or an unknown function to be learnt. The state *s*(*t*) is then the count of cardiac cycles since the model was initialized (with *s*(0) = 0), and *s*_*w*_(*t*) varies between 0 and 1 throughout each cardiac cycle.^1^ Equation (2.4) is then replaced with equation (2.33), as long as the parameters ${\hat{A}}_{i},{\hat{B}}_{i},{\hat{C}}_{i}$ are tuned for a cardiac period of 1 s. Note that this transformation is not limited to just sums of exponentials: the same technique could be used to transform any arbitrary constant HR *e*(*t*) into a variable HR *e*(*s*_*w*_). The new HR model is then given by

2.31 $$\frac{ds}{dt}=\frac{\mathrm{HR}(t)}{60},$$

2.32 $$s_{w}(t)=s(t)\;\mathrm{mod}\;1$$

2.33 $$\mathrm{and}\;\;\;e(s_{w})=\sum_{i=1}^{N}{\hat{A}}_{i}e^{-{\hat{B}}_{i}{\left(s_{w}-{\hat{C}}_{i}\right)}^{2}}.$$

### 2.5. Model parameters and initial conditions

Full parametrizations for all biophysical models are included in tables 1 to 5. As detailed below, the parametrization is taken from previously published works, except where they are believed to be erroneous, as the contribution of this work is methodological in nature and does not depend on any specific novel parametrization. All parameters are converted into consistent units of pressure (mmHg) and volume (litres).

**Table 5.** Parameters for respiratory model [18]. Note that *R*_ua_, *R*_ca_ and *V*_th_, 0 are given with incorrect units in the original paper, and HB, *λ* and *μ* are given with no units. The parameter *β* is introduced in §2.3. Bear in mind that the Liènard system parameters are distinct from the similarly named *A*_*i*_, *B*_*i*_ in the cardiac driver function.

parameter	value
alveolar elastance (*E*_alv_)	3.678 mmHg l^−1^
chest wall elastance (*E*_cw_)	2.942 mmHg l^−1^
upper airways resistance (*R*_ua_)	3.678 mmHg s l^−1^
central airways resistance (*R*_ca_)	0.7356 mmHg s l^−1^
intrathoracic volume at zero pressure (*V*_th_, 0)	2 l
Hering–Breuer reflex constant (HB)	1 l^−1^
gain on Liènard output in *P*_mus_ derivative (*λ*)	1.5 mmHg s^−1^
derivative offset for *P*_mus_ (*μ*)	1 mmHg s^−1^
integral control gain on *P*_mus_ (*β*)	0.1 s^−1^
Liènard system parameter (*a*)	−0.8
Liènard system parameter (*b*)	−3

Complete and accurate parameter listings for the inertial cardiovascular model are rare in the existing published literature due to the challenges of *in situ* measurement and variations across animal models, species etc. One useful source is [1], but with a few necessary modifications: the given value for *E*_ao_ is 0, which is assumed to be a mistake as it results in non-physical behaviour, and *R*_pul_ and *R*_sys_ are missing. The values in tables 3 and 4 came from a later PhD thesis [49]. Furthermore, the referenced value of the offset parameter *C*_*i*_ in the cardiac driver function (equation (2.4)) appears to be incorrect, as setting it to anything other than half the cardiac cycle duration results in a discontinuity at the end of the cycle. Finally, *V*_tot_ is described as 5.5 l in the referenced source, but due to the low total dead-space volume in table 3, this results in non-physical behaviour, therefore a stressed blood volume of 1.5 l is assumed (and the model ignores the 4 l of dead space). Another solution would have been to restore the dead space in the PV compartments, which sum to 4 l (excluding the virtual septum volume).

Table 5 gives Jallon’s parameters for the respiratory model. In tables 1 and 2, there are some alternative parameter values provided by Jallon [18] in brackets which reduce the stiffness of the septum; these are described as being necessary to achieve reasonable physiological values for all variables. However, no physical justification was made for this change, and they cause the ventricle PV loops (figure 2) to lose their shape completely. As a result, these parameters are not used in the simulations.

All initial state values are given in tables 6, 7 and 8. The initial compartment volumes must sum to *V*_tot_ (and this is the only place the parameter is used). Apart from this, the asymptotic behaviour of the model does not depend on the individual initial volumes, as long as they are sensible. The closer the initialization is to the asymptotic behaviour, the shorter the initial transient period will be. Alternatively, initialization could be performed by an optimization procedure [29].

**Table 6.** Initial volumes for non-inertial cardiovascular system model parametrized by tables 1 and 2.

state	initial value (l)
pulmonary artery volume (*V*_pa_)	0.187
pulmonary vein volume (*V*_pu_)	0.902
left ventricle volume (*V*_lv_)	0.1375
right ventricle volume (*V*_rv_)	0.132
aorta volume (*V*_ao_)	0.9515
vena cava volume (*V*_vc_)	3.190

**Table 7.** Initial volumes for inertial cardiovascular system model parametrized by tables 3 and 4. Initial flow rates can be calculated using equation (2.15). Note that the unstressed blood volume is not considered in this parametrization.

state	initial value (l)
pulmonary artery volume (*V*_pa_)	0.043
pulmonary vein volume (*V*_pu_)	0.808
left ventricle volume (*V*_lv_)	0.095
right ventricle volume (*V*_rv_)	0.091
aorta volume (*V*_ao_)	0.133
vena cava volume (*V*_vc_)	0.330

**Table 8.** Initial volume ratios for respiratory model parametrized by table 5. The Liènard system states are both dimensionless.

state	initial value
Liènard system state (*x*)	−0.6
Liènard system state (*y*)	0
respiratory muscular pressure (*P*_mus_)	0 mmHg

The models were simulated using Diffrax [31] with the Tsit5 solver, frequently recommended as a good general-purpose ODE solver [31,50,51]. Adaptive step sizing was used, with the default proportional error control; this is important due to the discontinuities introduced by the valves, which require a smaller step size to resolve than the rest of the solution. The absolute tolerance was set to 10^−7^ as the unwanted negative flow rates described in §2.1.3 were found to respond linearly to this with a gain of roughly 100, giving typical values of around −10^−5^ 1 s^−1^. The relative tolerance was set to 10^−4^, as with the default setting of 10^−3^, the interpolated dense solution for the non-inertial model was noticeably inaccurate around the closing of the pulmonary and aortic valves, with oscillating flow rates between ODE steps.

## 3. Results

Results of simulating the various models presented in §2 are shown below. The dense ODE solution is interpolated onto a regular grid, so that a maximum step size does not need to be set just for visualization purposes. States were expressed in units of litres (but converted to millilitres for plotting), and pressures were calculated in mmHg. Figure 3 shows two seconds of simulation for the inertial and non-inertial Smith CVS models with ventricular interaction, using parameters from tables 3 and 4 in both cases. No previous published comparison of these two models with matching parameters has been found. In the upper right corner of the PV diagram, the inertial model is less rounded due to the sharper ejection profile, which is also visible in *Q*_av_ and *Q*_pv_. Other than this, both models are very similar; in particular, the septum deviation volume *V*_spt_ is almost unaffected.

**Figure 6.**
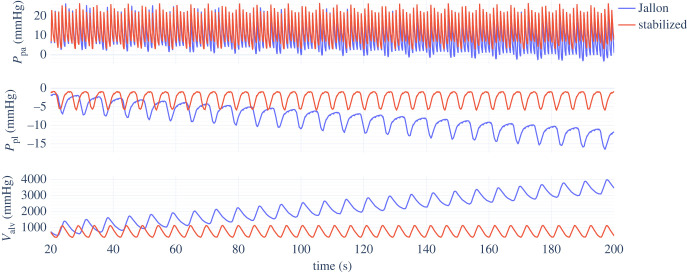
Selected outputs from the Jallon heart-lungs model, with parameters from tables 1, 2 and 5. The original behaviour (*β* = 0) is plotted in blue, with the stabilized model (*β* = 0.1) overlaid in red. Note the gradual drift in behaviour.

Figure 6 shows the behaviour of the Jallon heart-lung model presented in §2.2 [18]. Following the original implementation, the linearization of *V*_spt_ is used, although the suggested parameter modifications (such as reducing the stiffness of the septum) were discarded, as explained in §2.5. The behaviour matches expectations, with left ventricle stroke volume decreasing during inspiration and increasing during expiration, and vice versa for the right ventricle. However, a gradual drift in almost all outputs can be seen, due to a growth of *P*_mus_ over time.

**Figure 7.**
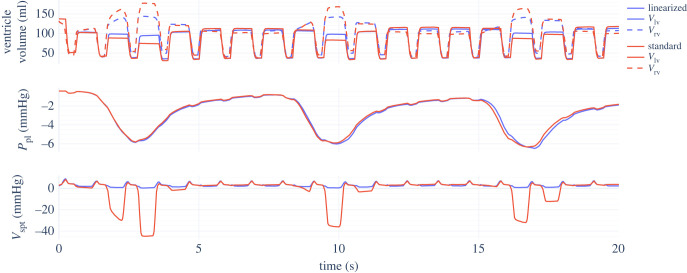
Comparison of standard nonlinear and linearized models of ventricular interaction, as components of the Jallon heart-lungs model with parameters from tables 1, 2 and 5 with *β* = 0.

Figure 7 gives a clear comparison of the effects of Jallon’s linearization of the ventricular interaction. Although the linearization has little influence on the numerical results during periods of static pleural pressure (or when the lungs are mostly exhaled), there is a noticeable difference during diastole immediately after inhalation. The full nonlinear model suggests a substantial septal deflection into the left ventricle in this situation, but the linearized model does not show this.

**Figure 8.**
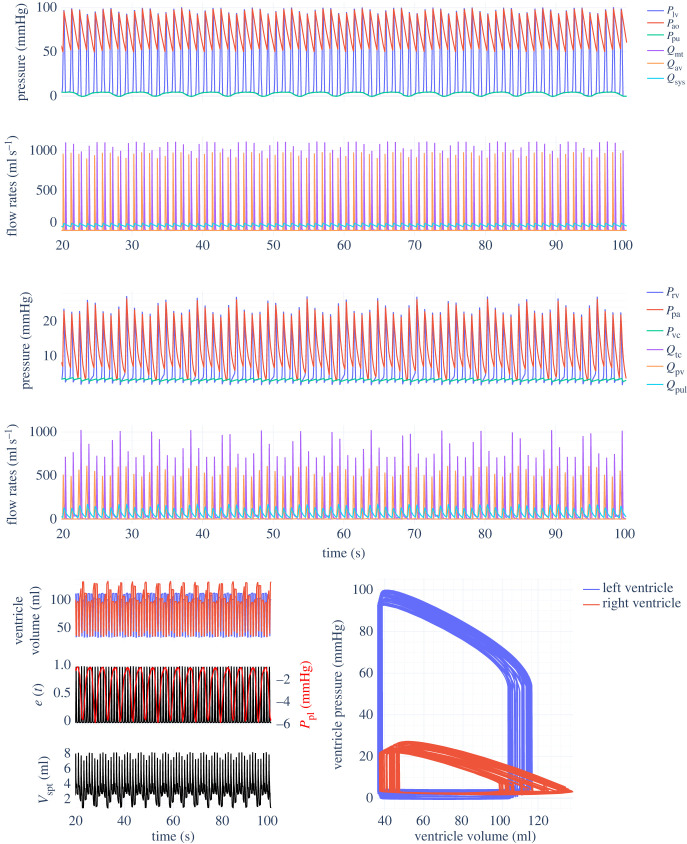
Jallon heart-lungs model outputs with parameters from tables 1, 2 and 5 with *β* = 0.1. Note the stable behaviour over time.

The results of the correction proposed in §2.3 that intends to stabilize Jallon’s respiratory model are shown in figure 8, confirming that there is no longer any drift over time. The HB parameter has been set to zero as the reflex has been accounted for by setting *β* = 0.1. The linearized ventricular interaction model was retained for a direct comparison against the original model.

**Figure 9.**
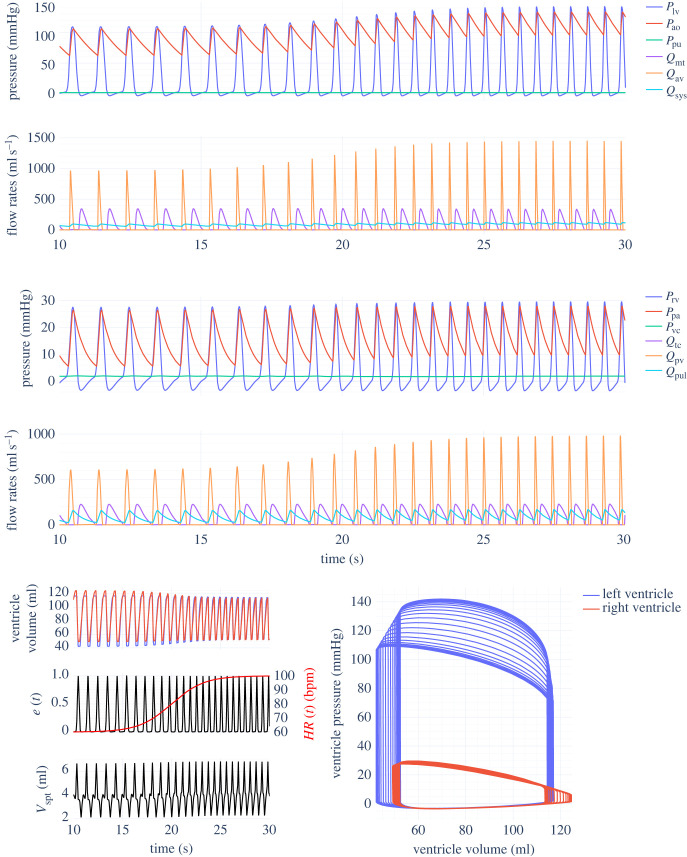
Smith inertial cardiovascular system model outputs with parameters from tables 3 and 4. Variable heart rate simulated with model from §2.4 and equation (3.1).

Figure 9 shows the results of the inertial Smith model with the variable HR extension proposed in §2.4. As the HR gradually increases from 60 up to 100, following

3.1 HR(t)=80+20tanh⁡(0.3(t−20)),

the peak flow rates through the aortic and pulmonary valves increase, and systolic/diastolic arterial pressures all increase substantially. The full nonlinear *V*_spt_ solver (using equation (2.12)) was used in this simulation.

Table 9 shows the computation time achieved in JAX for each of the model configurations simulated.

**Table 9.** Computation time in JAX for 60 s of simulation (not including compilation time), averaged over 10 runs. Run on an AMD Ryzen 7 5800H CPU in Windows Subsystem for Linux. Linear/nonlinear refers to ventricular interaction; the Jallon model is always non-inertial. The compiled-in max number of steps in Diffrax was set to 16^4^.

configuration	computation time	steps
Smith non-inertial	58 ms	6000
Smith inertial	110 ms	19 927
Smith non-inertial, variable HR	67 ms	6307
Jallon (linear)	16 ms	7062
Jallon (nonlinear)	64 ms	7060
Jallon (linear, stabilized)	16 ms	6982

## 4. Discussion

It is difficult to evaluate the accuracy of the results presented in §3 in comparison with the published versions, due to the lack of provided code and occasionally insufficient descriptions of the parameters. However, qualitative comparison with the available plots suggests that all models were correctly reproduced. Upon inhalation, right ventricle stroke volume increases and left ventricle decreases, as expected.

The *V*_spt_ linearization defined by equation (2.14) had a much larger than expected effect on the *V*_spt_ trace when combined with the Jallon model. During inspiration, it results in a substantial under-prediction of septal deflection compared with the full nonlinear model. Recall that the linearization is motivated by computational efficiency rather than accuracy, so regardless of which is more physically accurate, the intention of the model has been substantially affected. However, the criteria that the *V*_spt_ variation should be around 4% of the ventricle, quoted in Smith’s original work [14,34], is no longer met in the Jallon model with nonlinear ventricular interaction, and the right ventricle expands notably. If the septal elasticity were further reduced, as suggested by Jallon, this effect would be even more pronounced.

The computational advantages of the linearized ventricular interaction were found here to be limited. In an earlier PyTorch implementation, the nonlinear Newton–Raphson solver already converged within one or two iterations when initialized to the value of the last accepted ODE step (so the improvement was negligible). In the final JAX implementation, varying the initial guess was not found to be possible. Despite this, even though the linearization resulted in a speed-up of over three times, the model was already capable of simulating one second of activity in under 1 ms. However, the linearization may be useful in a parameter estimation setting where the simulation might need to be iterated, and the backwards pass may also be sped up with the linearization.

The modification proposed in §2.3 to stabilize the respiratory model is considered to be successful, and is necessary for long-term simulations of the Jallon model. The modification does not affect the physical relevance of the model, as it only slightly adjusts the use of the Liènard system as a signal generator.

The variable HR model proposed in §2.4 also works as intended, allowing for ICU HR data to be fed directly into the CVS models. This change would be useful when performing parameter estimation on long periods of patient data, as it provides an alternative to the beat-to-beat approach [52]. On the other hand, the physiological relevance of the model results remain to be verified. Figure 9 suggests that when the HR increases, the diastolic aortic pressure should noticeably increase, with the systolic pressure also increasing but at a shallower rate. However, studies on patients fitted with pacemakers suggest that in humans, although the diastolic aortic pressure does indeed increase when the HR is artificially raised (as do peripheral systolic and diastolic arterial pressures), the systolic aortic pressure does not increase [53,54]. This effect is understood to be due to wave reflection and change in systemic arterial stiffness, mechanisms that are not simulated within this model framework. Such neglected aspects go some way to explaining the difference in behaviour between the model and experimental results.

There are a wide range of mechanisms for the autonomous regulation of blood pressure [55] that are not considered by this model. Furthermore, an assumption is (most likely incorrectly) made that the length of systole as a proportion of the cardiac period does not change with HR, even when the driving pulse (equation (2.33)) is squashed or stretched in time depending on the rate of change of *s*_*w*_(*t*). Finally, the shape of the cardiac driver function used within this model is extremely simplistic, being defined by a single parameter alone (${\hat{B}}_{1}$). It would in theory be possible to redefine this parameter to be a function of HR; this approach could also be extended to more complicated driver functions [34–36,56].

Although it is not demonstrated in this work, the use of an ODE solver that supports autodifferentiation may permit the parameters to be learned directly by minimizing a loss versus clinically measured patient-specific blood pressure and HR data. Furthermore, it could lead to a grey-box model that combines human-designed models of the CVS with regulated variables such as HR, elastances and resistances implemented as unknown functions (of time and/or state variables) to be learned, for example neural ODEs [38]. Such models would be valuable as they could estimate information about a patient that would be infeasible or overly intrusive to measure directly. In particular, this approach of combining a simple model with learned functions would address the issue of increasingly complex models having many unknown physiological parameters which cannot be uniquely identified from commonly available patient data. In conjunction with real-time data available in an ICU, this could provide vital extra information when making important decisions on treatments.

## 5. Conclusion

Two novel modifications to the CVS models of Smith [14] and Jallon [18] were proposed: a correction to stabilize Jallon’s model (§2.3) and an extension to allow all models to simulate a variable HR (§2.4). Both modifications work as intended and significantly extend the capabilities of Smith’s original model.

The stabilization of the Jallon model is crucial to enable realistic simulation of, and parameter estimation from, physiological behaviour over clinically relevant time scales, and supersedes the previous attempt to incorporate the Hering–Breur reflex into the original model.

The novel approach to simulating variable HR, although applied here on a very simple model and basic cardiac driver function, has also been successful. However, the model suggests an increase in central systolic arterial pressure with increasing HR, which is not in agreement with available experimental data. It is postulated that this discrepancy may be due to the model neglecting the effects of pressure wave reflection and changes in systemic arterial stiffness with HR. On the other hand, the extension is generalizable to many other ODE models of the CVS, so a similar modification to a more complicated original model might yield more accurate results. For example, the CircAdapt [26] model might also be extended in this manner.

With the HR defined by an arbitrary function of time, the model is no longer limited to periodic behaviour. In conjunction with the support for autodifferentiation due to the implementation in JAX, this leads to further opportunities for applying the CVS model in conjunction with machine-learning techniques to measured bio-signals from patients, especially when considering the high computational cost of parameter estimation with models which do not support differentiation and have higher parameter counts [57].

## Data Availability

The code developed for this work is available from the GitHub digital repository: slishak/cvsx. An earlier version using PyTorch instead of JAX can also be found from the GitHub repository: slishak/MedicalTimeSeriesPrediction.

## Endnote

1 This could also be achieved using an ODE event solver, detecting the event *s*(*t*_*e*_) = 1 and resetting *s*(*t*_*e*_) := 0 before continuing, therefore having *s*_*w*_(*t*) = *s*(*t*) instead of equation (2.32). This would be preferable if having an ODE state that grows indefinitely with time is undesirable, for example as the error of *s*(*t*) can be better controlled using a relative tolerance if the magnitude of *s*(*t*) is bounded, or that if the model were used as a patient’s ‘digital twin’, the precision of *s*(*t*) would degrade over long periods of time due to floating point representation.

## References

[1] Paeme S et al. 2011 Mathematical multi-scale model of the cardiovascular system including mitral valve dynamics: application to ischemic mitral insufficiency. Biomed. Eng. Online **10**, 86. (10.1186/1475-925X-10-86) 21942971 PMC3271239

[2] Shi Y, Lawford P, Hose R. 2011 Review of zero-D and 1-D models of blood flow in the cardiovascular system. Biomed. Eng. Online **10**, 33. (10.1186/1475-925X-10-33) 21521508 PMC3103466

[3] Quarteroni A, Formaggia L. 2004 Mathematical modelling and numerical simulation of the cardiovascular system, computational models for the human body. Handbook Numer. Anal. **12**, 3-127. (10.1016/S1570-8659(03)12001-7)

[4] Matthys KS, Alastruey J, Peiró J, Khir AW, Segers P, Verdonck PR, Parker KH, Sherwin SJ. 2007 Pulse wave propagation in a model human arterial network: assessment of 1-D numerical simulations against in vitro measurements. J. Biomech. **40**, 3476-3486. (10.1016/j.jbiomech.2007.05.027) 17640653

[5] Xiu D, Sherwin SJ. 2007 Parametric uncertainty analysis of pulse wave propagation in a model of a human arterial network. J. Comput. Phys. **226**, 1385-1407. (10.1016/j.jcp.2007.05.020)

[6] Doost SN, Zhong L, Su B, Morsi YS. 2017 Two-dimensional intraventricular flow pattern visualization using the image-based computational fluid dynamics. Comput. Methods Biomech. Biomed. Engin. **20**, 492-507. (10.1080/10255842.2016.1250891) 27796137

[7] Morris PD et al. 2016 Computational fluid dynamics modelling in cardiovascular medicine. Heart **102**, 18-28. (10.1136/heartjnl-2015-308044) 26512019 PMC4717410

[8] Sagawa K, Lie RK, Schaefer J, Frank O. 1990 Translation of Otto Frank’s paper ‘Die Grundform des Arteriellen Pulses’ Zeitschrift für Biologie 37: 483–526 (1899). J. Mol. Cell Cardiol. **22**, 253-254. (10.1016/0022-2828(90)91459-K) 2192068

[9] Parker KH. 2009 A brief history of arterial wave mechanics. Med. Biol. Eng. Comput. **47**, 111-118. (10.1007/s11517-009-0440-5) 19198914 PMC2644374

[10] Landes G. 1943 Einige untersuchungen an elektrischen analogieschaltungen zum kreitslaufsystem. Z. Biol. **101**, 418-429.

[11] Westerhof N, Elzinga G, Sipkema P. 1971 An artificial arterial system for pumping hearts. J. Appl. Physiol. **31**, 776-781. (10.1152/jappl.1971.31.5.776) 5117196

[12] Guyton AC, Coleman TG, Granger HJ. 1972 Circulation: overall regulation. Annu. Rev. Physiol. **34**, 13-44. (10.1146/annurev.ph.34.030172.000305) 4334846

[13] Garber L, Khodaei S, Keshavarz-Motamed Z. 2022 The critical role of lumped parameter models in patient-specific cardiovascular simulations. Arch. Comput. Methods Eng. **29**, 2977-3000. (10.1007/s11831-021-09685-5)

[14] Smith BW, Chase JG, Nokes RI, Shaw GM, Wake G. 2004 Minimal haemodynamic system model including ventricular interaction and valve dynamics. Med. Eng. Phys. **26**, 131-139. (10.1016/j.medengphy.2003.10.001) 15036180

[15] Lu K, Clark Jr J, Ghorbel F, Ware D, Bidani A. 2001 A human cardiopulmonary system model applied to the analysis of the Valsalva maneuver. Am. J. Physiol.-Heart Circul. Physiol. **281**, H2661-H2679. (10.1152/ajpheart.2001.281.6.H2661) 11709436

[16] Bai J, Lu H, Zhang J, Zhao B, Zhou X. 1998 Optimization and mechanism of step–leap respiration exercise in treating of cor pulmonale. Comput. Biol. Med. **28**, 289-307. (10.1016/S0010-4825(98)00009-2) 9784965

[17] De Lazzari C, Darowski M, Ferrari G, Pisanelli DM, Tosti G. 2006 Modelling in the study of interaction of Hemopump device and artificial ventilation. Comput. Biol. Med. **36**, 1235-1251. (10.1016/j.compbiomed.2005.08.001) 16202402

[18] Fontecave-Jallon J, Abdulhay E, Calabrese P, Baconnier P, Gumery PY. 2009 A model of mechanical interactions between heart and lungs. Phil. Trans. R. Soc. A **367**, 4741-4757. (10.1098/rsta.2009.0137) 19884178

[19] Fernandes LG, Trenhago PR, Feijóo RA, Blanco PJ. 2021 Integrated cardiorespiratory system model with short timescale control mechanisms. Int. J. Numer. Methods Biomed. Eng. **37**, e3332. (10.1002/cnm.3332) 32189436

[20] Trenhago PR, Fernandes LG, Müller LO, Blanco PJ, Feijéo RA. 2016 An integrated mathematical model of the cardiovascular and respiratory systems. Int. J. Numer. Method Biomed. Eng. **32**, e02736. (10.1002/cnm.2736) 26198626

[21] Albanese A, Cheng L, Ursino M, Chbat NW. 2016 An integrated mathematical model of the human cardiopulmonary system: model development. Am. J. Physiol.-Heart Circul. Physiol. **310**, H899-H921. (10.1152/ajpheart.00230.2014) 26683899

[22] Smith BW, Andreassen S, Shaw GM, Jensen PL, Rees SE, Chase JG. 2007 Simulation of cardiovascular system diseases by including the autonomic nervous system into a minimal model. Comput. Methods Programs Biomed. **86**, 153-160. (10.1016/j.cmpb.2007.02.001) 17350711

[23] Ursino M. 1998 Interaction between carotid baroregulation and the pulsating heart: a mathematical model. Am. J. Physiol.-Heart Circul. Physiol. **275**, H1733-H1747. (10.1152/ajpheart.1998.275.5.H1733) 9815081

[24] Jin X, Laxminarayan S, Nagaraja S, Wallqvist A, Reifman J. 2003 Development and validation of a mathematical model to simulate human cardiovascular and respiratory responses to battlefield trauma. Int. J. Numer. Methods Biomed. Eng. **39**, e3662. (10.1002/cnm.3662) 36385572

[25] Sarmiento CA, Hernández AM, Serna LY, Mañanas MÁ. 2021 An integrated mathematical model of the cardiovascular and respiratory response to exercise: model-building and comparison with reported models. Am. J. Physiol. Heart Circ. Physiol. **320**, H1235-H1260. (10.1152/ajpheart.00074.2020) 33416450

[26] Arts T, Delhaas T, Bovendeerd P, Verbeek X, Prinzen FW. 2005 Adaptation to mechanical load determines shape and properties of heart and circulation: the CircAdapt model. Am. J. Physiol. Heart Circ. Physiol. **288**, H1943-H1954. (10.1152/ajpheart.00444.2004) 15550528

[27] Arts T, Lumens J, Kroon W, Delhaas T. 2012 Control of whole heart geometry by intramyocardial mechano-feedback: a model study. PLoS Comput. Biol. **8**, 1-12. (10.1371/journal.pcbi.1002369) 22346742 PMC3276542

[28] Walmsley J, Arts T, Derval N, Bordachar P, Cochet H, Ploux S, Prinzen FW, Delhaas T, Lumens J. 2015 Fast simulation of mechanical heterogeneity in the electrically asynchronous heart using the multipatch module. PLoS Comput. Biol. **11**, 1-23. (10.1371/journal.pcbi.1004284) PMC4512705 26204520

[29] Smith BW. 2004 Minimal haemodynamic modelling of the heart & circulation for clinical application. PhD thesis, University of Canterbury, Christchurch, New Zealand.

[30] Bradbury J, Frostig R, Hawkins P, Johnson MJ, Leary C, Maclaurin D, Necula G, Paszke A, VanderPlas J, Wanderman-Milne S, Zhang Q. 2018 JAX: composable transformations of Python+NumPy programs, version 0.3.13. See https://github.com/google/jax.

[31] Kidger P. 2021 On neural differential equations. PhD thesis, University of Oxford, UK.

[32] Smith BW, Geoffrey Chase J, Shaw GM, Nokes RI. 2005 Experimentally verified minimal cardiovascular system model for rapid diagnostic assistance. Control Eng. Practice **13**, 1183-1193. (10.1016/j.conengprac.2004.10.014)

[33] Suga H, Sagawa K, Shoukas AA. 1973 Load independence of the instantaneous pressure-volume ratio of the canine left ventricle and effects of epinephrine and heart rate on the ratio. Circ. Res. **32**, 314-322. (10.1161/01.RES.32.3.314) 4691336

[34] Chung DC, Niranjan SC, Clark JW, Bidani A, Johnston WE, Zwischenberger JB, Traber DL. 1997 A dynamic model of ventricular interaction and pericardial influence. Am. J. Physiol. **272**, H2942-H2962. (10.1152/ajpheart.1997.272.6.H2942) 9227574

[35] Starfinger C. 2008 Patient-specific modelling of the cardiovascular system for diagnosis and therapy assistance in critical care. PhD thesis, University of Canterbury, Christchurch, New Zealand.

[36] Stevenson D. 2013 Estimation of the time-varying elastance of the left and right ventricles. PhD thesis, University of Canterbury, Christchurch, New Zealand.

[37] Hann CE, Chase JG, Shaw GM. 2005 Efficient implementation of non-linear valve law and ventricular interaction dynamics in the minimal cardiac model. Comput. Methods Programs Biomed. **80**, 65-74. (10.1016/j.cmpb.2005.06.003) 16039750

[38] Chen RTQ, Rubanova Y, Bettencourt J, Duvenaud DK. 2018 Neural ordinary differential equations. In *Advances in neural information processing systems* (eds S Bengio, H Wallach, H Larochelle, K Grauman, N Cesa-Bianchi, R Garnett), vol. 31. Red Hook, NY: Curran Associates, Inc.

[39] Kasim MF, Vinko SM. 2020 *ξ*-torch: differentiable scientific computing library. *arXiv*. (http://arxiv.org/abs/2010.01921)

[40] Smith BW, Chase JG, Nokes RI, Shaw GM, David T. 2003 Velocity profile method for time varying resistance in minimal cardiovascular system models. Phys. Med. Biol. **48**, 3375-3387. (10.1088/0031-9155/48/20/008) 14620064

[41] Hairer E, Nørsett S, Wanner G. 2008 Solving ordinary differential equations I: nonstiff problems, 2nd revised edn. Berlin, Germany: Springer.

[42] Liénard A. 1928 Etude des oscillations entretenues. Revue Generale de l’Elactricite **23**, 901-902.

[43] Knox CK. 1973 Characteristics of inflation and deflation reflexes during expiration of the cat. J. Neurophysiol. **36**, 284-295. (10.1152/jn.1973.36.2.284) 4706263

[44] Clark FJ, von Euler C. 1972 On the regulation of depth and rate of breathing. J. Physiol. **222**, 267-295. (10.1113/jphysiol.1972.sp009797) 5033464 PMC1331381

[45] Gaudenzi F, Avolio AP. 2013 Lumped parameter model of cardiovascular-respiratory interaction. Annu. Int. Conf. IEEE Eng. Med. Biol. Soc. **2013**, 473-476. (10.1109/EMBC.2013.6609539) 24109726

[46] Fontecave-Jallon J, Baconnier P. 2016 A simple mathematical model of spontaneous swallow effects on breathing based on new experimental data. In *2016 38th Annual Int. Conf. of the IEEE Engineering in Medicine and Biology Society (EMBC)*, pp. 4260–4263. (10.1109/EMBC.2016.7591668).

[47] West J, Luks A. 2021 West’s respiratory physiology: the essentials, 11th edn. Philadelphia, PA: Wolters Kluwer.

[48] Ang KH, Chong G, Li Y. 2005 PID control system analysis, design, and technology. IEEE Trans. Control Syst. Technol. **13**, 559-576. (10.1109/TCST.2005.847331)

[49] Revie JAM. 2012 Model-based cardiovascular monitoring in critical care for improved diagnosis of cardiac dysfunction. PhD thesis, University of Canterbury, Christchurch, New Zealand.

[50] Tsitouras C. 2011 Runge–Kutta pairs of order 5(4) satisfying only the first column simplifying assumption. Comput. Math. Appl. **62**, 770-775. (10.1016/j.camwa.2011.06.002)

[51] Rackauckas C, Nie Q. 2017 DifferentialEquations.jl – a performant and feature-rich ecosystem for solving differential equations in Julia. J. Open Res. Softw. **5**, 15. (10.5334/jors.151)

[52] Stevenson D, Revie J, Chase JG, Hann CE, Shaw GM, Lambermont B, Ghuysen A, Kolh P, Desaive T. 2012 Beat-to-beat estimation of the continuous left and right cardiac elastance from metrics commonly available in clinical settings. Biomed. Eng. Online **11**, 73. (10.1186/1475-925X-11-73) 22998792 PMC3538613

[53] Wilkinson IB, MacCallum H, Flint L, Cockcroft JR, Newby DE, Webb DJ. 2000 The influence of heart rate on augmentation index and central arterial pressure in humans. J. Physiol. **525**, 263-270. (10.1111/j.1469-7793.2000.t01-1-00263.x) 10811742 PMC2269933

[54] Wilkinson IB, Mohammad NH, Tyrrell S, Hall IR, Webb DJ, Paul VE, Levy T, Cockcroft JR. 2002 Heart rate dependency of pulse pressure amplification and arterial stiffness. Am. J. Hypertens. **15**, 24-30. (10.1016/S0895-7061(01)02252-X) 11824855

[55] Shahoud JS, Sanvictores T. 2022 Physiology, arterial pressure regulation. In StatPearls (ed. NR Aeddula). Treasure Island, FL: StatPearls Publishing. 30860744

[56] Hann CE et al. 2011 Patient specific identification of the cardiac driver function in a cardiovascular system model. Comput. Methods Programs Biomed. **101**, 201-207. (10.1016/j.cmpb.2010.06.005) 20621383

[57] van Osta N et al. 2020 Parameter subset reduction for patient-specific modelling of arrhythmogenic cardiomyopathy-related mutation carriers in the CircAdapt model. Phil. Trans. R. Soc. A **378**, 20190347. (10.1098/rsta.2019.0347) 32448061 PMC7287326

[58] CHIMERA, About us, https://www.ucl.ac.uk/chimera/about-us (visited on 08 October 2022).

